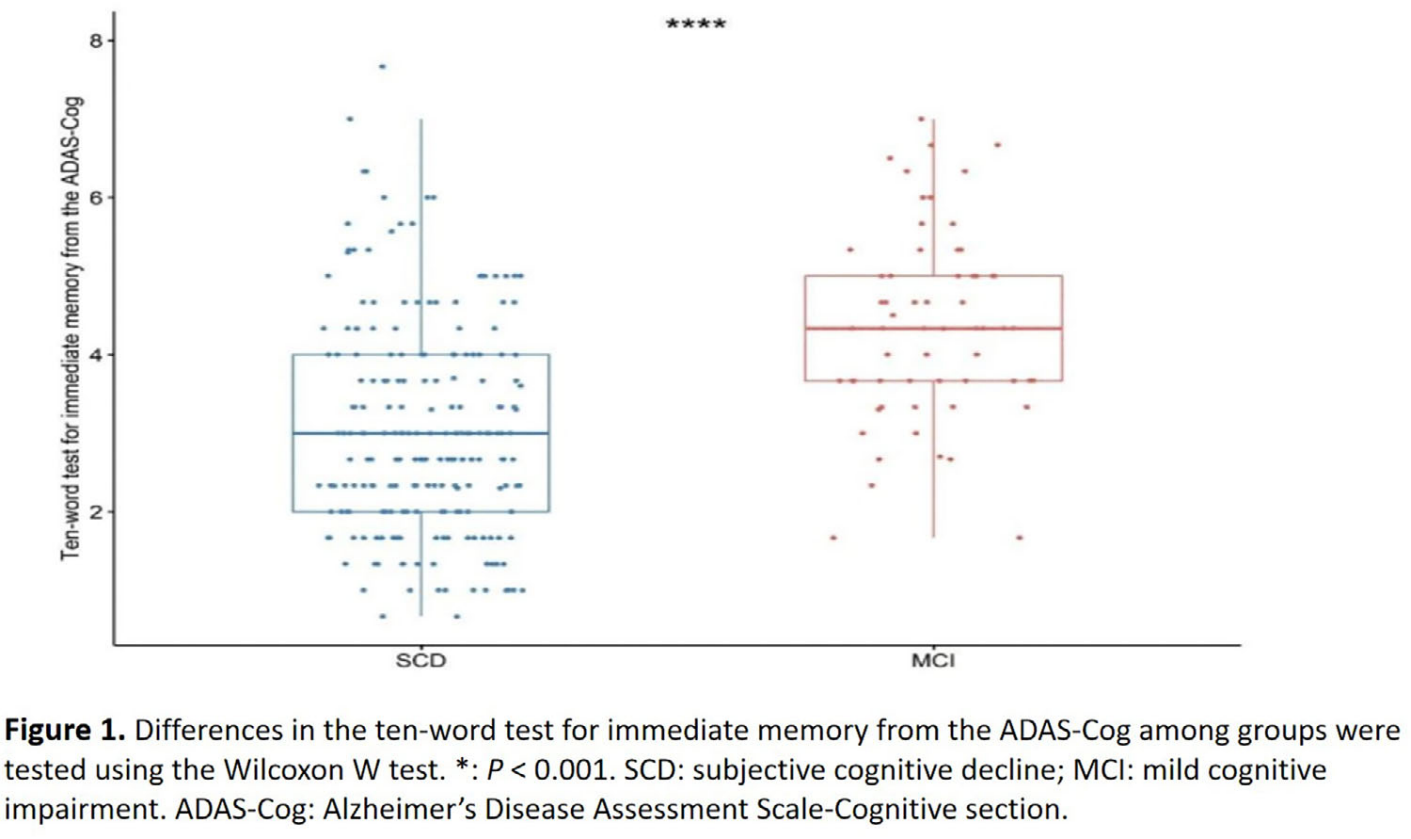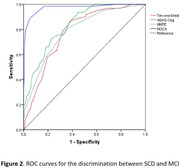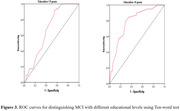# Ten‐word Test:An Effective Tool to Differential Mild Cognitive Impairment from Subjective Cognitive Decline

**DOI:** 10.1002/alz.091251

**Published:** 2025-01-03

**Authors:** Hua Ren, LI Dong, Tiejun Liu, Ziqi Wang

**Affiliations:** ^1^ The Clinical Hospital of Chengdu Brain Science Institute, Chengdu, Sichuan China; ^2^ University of Electronic Science and Technology of China, Chengdu, Sichuan China; ^3^ The Clinical Hospital of Chengdu Brain Science Institute, MOE Key Lab for Neuroinformation, School of Life Science and Technology, University of Electronic Science and Technology of China, Chengdu China

## Abstract

**Background:**

Identifying the transition from subjective cognitive decline (SCD) to mild cognitive impairment (MCI) is crucial for delaying the progression of dementia and enabling early intervention. In clinical practice, there are many deficiencies in the scale tools used to identify the two. We wanted to evaluate the application of the 10‐word test in identifying SCD and MCI.

**Methods:**

A total of 62 MCI and 203 SCD subjects were assessed and underwent multiple neuropsychological assessment, including the Alzheimer’s Disease Assessment Scale‐Cognitive Subscale (ADAS‐cog), Mini‐Mental State Examination (MMSE), Montreal Cognitive Assessment‐B (MOCA‐B), and Activities of Daily Living scale (ADL). We performed a statistical analysis on these assessments results.

**Results:**

Neuropsychological assessment results of the MCI and SCD groups indicated significant differences in the scores of the ten‐word test (*P*< 0.001, figure 1), with the MCI group scoring higher. When the cut‐off value for the ten‐word test was set at 3.15, the sensitivity for differentiating MCI from SCD was 87%, and specificity was 61% (AUC 0.777, *P*< 0.001, figure 2). DeLong’s test revealed no statistically significant difference in the ability of the ten‐word test to distinguish between MCI and SCD compared to the total score of ADAS‐Cog (AUC 0.833) and MMSE (AUC 0.784) (*P* > 0.05), but a significant difference was observed when compared to MOCA (AUC 0.973, *P* < 0.001). In the population with an education level of ≤ 9 years, the optimal cut‐off value for the ten‐word test was 3.15, with a sensitivity of 91% and specificity of 45% (AUC = 0.674, *P* = 0.030, figure 3). In the population with an education level of > 9 years, the optimal cut‐off value was 3.63, with a sensitivity of 79% and specificity of 71% (AUC = 0.785, *P* < 0.001, figure 3).

**Conclusion:**

Memory impairment is the most common complaint in MCI and SCD. The 10‐word immediate recall test from the ADAS‐cog may objectively reflect the short‐term memory level of subjects, with its simplicity and quick administration. It serves as an effective and convenient tool for rapid identification of mild cognitive impairment.